# Ageism, Healthy Life Expectancy and Population Ageing: How Are They Related?

**DOI:** 10.3390/ijerph17093159

**Published:** 2020-05-01

**Authors:** Alana Officer, Jotheeswaran Amuthavalli Thiyagarajan, Mira Leonie Schneiders, Paul Nash, Vânia de la Fuente-Núñez

**Affiliations:** 1 Department of Ageing and Life Course, World Health Organization, 20 Avenue Appia, 1221 Geneva, Switzerland; officera@who.int (A.O.); amuthavallithiya@who.int (J.A.T.); 2 Ethox Centre, Nuffield Department of Population Health, University of Oxford, Oxford OX3 7LF, UK; mira.schneiders@gtc.ox.ac.uk; 3 Leonard Davis School of Gerontology, University of Southern California, Los Angeles, CA 90089, USA; pnash@usc.edu

**Keywords:** ageism, prevalence, healthy life expectancy, age stereotypes, age prejudice

## Abstract

Evidence shows that ageism negatively impacts the health of older adults. However, estimates of its prevalence are lacking. This study aimed to estimate the global prevalence of ageism towards older adults and to explore possible explanatory factors. Data were included from 57 countries that took part in Wave 6 of the World Values Survey. Multilevel Latent Class Analysis was performed to identify distinct classes of individuals and countries. Individuals were classified as having high, moderate or low ageist attitudes; and countries as being highly, moderately or minimally ageist, by aggregating individual responses. Individual-level (age, sex, education and wealth) and contextual-level factors (healthy life expectancy, population health status and proportion of the population aged over 60 years) were examined as potential explanatory factors in multinomial logistic regression. From the 83,034 participants included, 44%, 32% and 24% were classified as having low, moderate and high ageist attitudes, respectively. From the 57 countries, 34 were classified as moderately or highly ageist. The likelihood of an individual or a country being ageist was significantly reduced by increases in healthy life expectancy and the proportion of older people within a country. Certain personal characteristics—younger age, being male and having lower education—were significantly associated with an increased probability of an individual having high ageist attitudes. At least one in every two people included in this study had moderate or high ageist attitudes. Despite the issue’s magnitude and negative health impacts, ageism remains a neglected global health issue.

## 1. Introduction

People worldwide are living longer. Between 2015 and 2050, the proportion of the world’s population over 60 years will nearly double from 12% (900 million people) to 22% (2 billion) [1]. The extent to and speed at which these demographic transitions are happening underscore the pressing need to develop appropriate responses to population ageing. However, commonly held perceptions and assumptions about older people and ageing pose serious challenges to developing an adequate societal response to population ageing. Older age is generally typecast as a period of frailty and inevitable decline in capacity, with the depiction of older people as a homogeneous group that is care dependent, burdensome on health and social care spending, and a hindrance to economic growth. This is inconsistent with the diversity in health and functioning that is seen in older age [2].

Ageism is the umbrella term for the stereotyping, prejudice and discrimination towards individuals on the basis of their chronological age or a perception of them as being “too old” or “too young” to be or do something [3]. Stereotypes affect how we think (cognition), prejudice affects how we feel (emotion), and discrimination affects how we act (behaviour) towards people on the basis of their age. Although ageism can affect any age group, it most often affects older people [4], and it is strongly institutionalised, generally accepted and unchallenged, largely because of its implicit and subconscious nature [5]. Ageist depictions are prevalent in everyday language and in the media [6]. Ageist policies such as health care rationing by age and institutional policies and practices that perpetuate stereotypical beliefs, such as mandatory retirement and the shortage of training programmes on ageing for health professionals, are widespread [7,8].

A growing body of research has examined the effects of ageism on the health and physical and cognitive functioning of older adults. For example, a meta-analysis found that direct exposure to negative ageing stereotypes was significantly associated with poorer performance on a range of physical and cognitive tasks in older people [9]. Once perceived as an “older adult”, not only do individuals become subjected to external stereotyping and discrimination but the ageist attitudes are internalised into unconscious self-stereotypes [10]. This internalisation process is significantly associated with poorer physical and mental health in older adults [11].

Despite an improved understanding of the negative consequences of ageism on the health and functioning of older people, there is limited understanding of the prevalence of this phenomenon and its relation to individual- and contextual-level factors. The few studies that have been conducted to study ageism prevalence across the world have used a maximum of two items to measure ageism [12,13]. This is problematic given the complex and multidimensional nature of this phenomenon. Other cross-national studies that have used a larger set of items to measure ageism had a limited geographical scope, covering one or two regions only [14,15].

To increase our knowledge in this area and to overcome the limitations of previous research, this study aimed to examine the global prevalence of ageism using a large population sample, nine different items and multi-level latent class analysis as the analytical strategy. It further aimed to study the association between ageism and a range of possible explanatory factors.

## 2. Materials and Methods

### 2.1. Data

The latest wave of the World Values Survey (WVS), Wave 6 (2010–2014), was the primary data source for this study. Fifty-seven of the countries (with a minimum of 1200 participants per country) that took part in this wave were included. Detailed information on the sampling method, survey administration and response rate in each country are reported elsewhere [16].

### 2.2. Dependent Variables

The primary dependent variable (ageism related attitudes) was determined from participants’ responses to nine questions from the WVS that relate to age and ageism and are similar to questions from existing ageism measures (e.g., the Fraboni Scale of Ageism and the Prescriptive Intergenerational-Tension Ageism Scale). Two of these questions asked participants to indicate how acceptable most people in their country would find it if (1) a suitably qualified 30-year-old was appointed as their boss, and (2) a suitably qualified 70-year-old was appointed as their boss. These two questions used a ten-point Likert scale ranging from one (completely unacceptable) to ten (completely acceptable). Three additional questions asked participants to indicate whether most people in their country viewed those aged over 70 as (3) friendly, (4) competent and (5) with respect. These three questions used a five-point Likert scale that ranged from zero (not at all likely to be viewed that way) to four (very likely to be viewed that way). The remaining four questions required participants to indicate whether they thought that (6) “older people are a burden on society”, (7) “older people get more than their fair share from the government”, (8) “companies that employ young people perform better than those that employ people of different ages”, and (9) “old people have too much political influence”. These four questions used a four-point Likert scale that ranged from one (strongly agree) to four (strongly disagree). Negatively worded questions were reverse coded. Therefore, high values for any of these questions indicated low ageist attitudes. For the analysis, the ten-point Likert responses were reclassified to form five-point Likert items ranging from one (unacceptable) to five (acceptable).

### 2.3. Individual-Level Variables

Individual-level variables included the following demographic and socioeconomic characteristics: age, sex, highest attainment in education and household income. “Education” was modelled as categorical in order to test non-linear effects and for consistency with the concept of education as a series of transitions between levels: no formal education or incomplete primary school, complete primary school, at least some secondary schooling, and university education (with or without a degree). Household income was measured using a self-rated income scale, with one representing the lowest and ten the highest income group.

### 2.4. Country-Level Variables

At the country level, the following explanatory variables were considered in the analysis: Healthy Life Expectancy (HLE), income inequality, self-reported health status at the population level, and the proportion of older people over 60 years of age. HLE was used as a summary measure of population health, and data on this measure were extracted from the Global Health Observatory database hosted by the World Health Organization [17]. Income inequality (Gini coefficient) data were taken from the United Nations Development Programme. A value of 0 represented complete equality, whereas a value of 1 represented absolute inequality [18]. Data on self-reported health status as reported in the WVS were used to estimate the proportion of the population in poor subjective health with data being imputed for each country to facilitate comparisons. Responses were coded in the WVS as “very good”, “good”, “fair”, “poor” or “very poor”. “Poor” and “very poor” responses were used in the analysis to estimate the proportion of individuals with poor subjective health. The proportion of the population aged 60 years and over in each country was taken from the 2015 United Nations demographic data [19]. Lastly, the World Bank’s income classification was used to classify countries by income level [20].

### 2.5. Statistical Analysis

All statistical analysis was performed using Mplus version 8 (Muthén & Muthén, Los Angeles, CA, USA) and STATA version 14 (Stata Corp LP, TX, USA). A Multilevel Latent Class Analysis (MLCA) using nonparametric estimation (i.e., not assuming normality) was performed to identify distinct latent classes of individuals and countries following the methodology described by Henry and Muthen [21]. Full information for class selection is provided in Supplementary Materials (Supplementary A). The effect of covariates on latent class membership was examined in multinomial logistic regression. To estimate the pooled prevalence of ageism by country income, a meta-analysis was carried out with proportions and 95% confidence intervals. The I^2^ value was used to quantify heterogeneity.

## 3. Results

The total number of participants was 83,034 (Table S1). The mean age of participants ranged from 31.5 to 53.3 years with 18% of the total sample aged 60 years and older. Highest educational attainment varied across countries, ranging from 12.3% of the sample in Zimbabwe to 86.4% in the United States of America. In the majority of countries, less than 20% of participants rated household income status as high. The average HLE of the countries included in the analysis was 65.1 years, ranging from 47.7 to 74.9 years, and the average proportion of people over 60 in these countries was 14% of the total population. The percentage of income inequality ranged from 8.8% to 57.3%, and the percentage of the population reporting to be in poor health ranged from 8% to 65% across countries.

In the multi-level latent class analysis, a model containing three latent classes of individuals—those with (i) high ageist attitudes, (ii) moderate ageist attitudes and (iii) low ageist attitudes—and three latent classes of countries—(i) highly ageist countries, (ii) moderately ageist countries and (iii) not very ageist countries—was identified as the best fit for the data based on inferential goodness-of-fit index in combination with several descriptive indices (see Table S2).

### 3.1. Individual Latent Classes of Ageism

**Figure 1:**
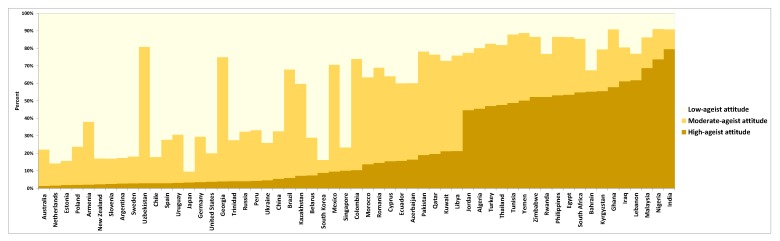
Distribution of individuals with high, moderate and low ageist attitudes by country.

**Figure 2:**
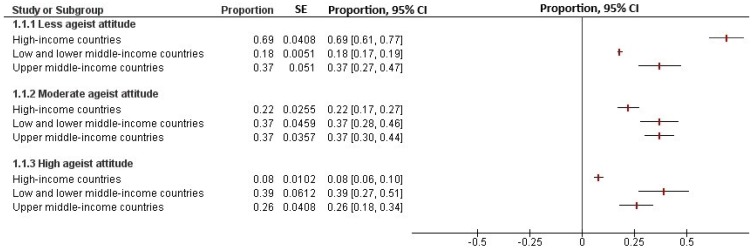
Meta-analysed estimates of individual-level ageist attitudes class by country income level.

From the 83,034 participants, 44%, 32% and 24% were classified as having low, moderate and high ageist attitudes, respectively. A close examination of these figures revealed that the distribution of individuals with high ageist attitudes was substantially higher in low- and middle-income countries (e.g., Nigeria, Bahrain and India), whereas the distribution of individuals with low ageist attitudes was higher in high-income countries (e.g., Poland, Australia and Japan) (see Figure 1). Meta-analysis further supported these results, showing that 39% (95% CI, 27% to 51%) of participants from low- and lower-middle-income countries present high ageist attitudes, whereas only 8% (95% CI, 6% to 10%) of participants from high-income countries present such attitudes. It also showed that as many as 69% (95% CI, 61% to 77%) of participants from high-income countries present low ageist attitudes whereas only 18% (95% CI, 17% to 19%) do so from low- and lower-middle-income countries (see Figure 2 and Figures S2–S10). The heterogeneity of these estimates was substantial (I^2^ = 99%; *p* < 0.001). See Figure S1 for the conditional response probabilities for the different ageism items. 

### 3.2. Country Latent Classes of Ageism

**Figure 3:**
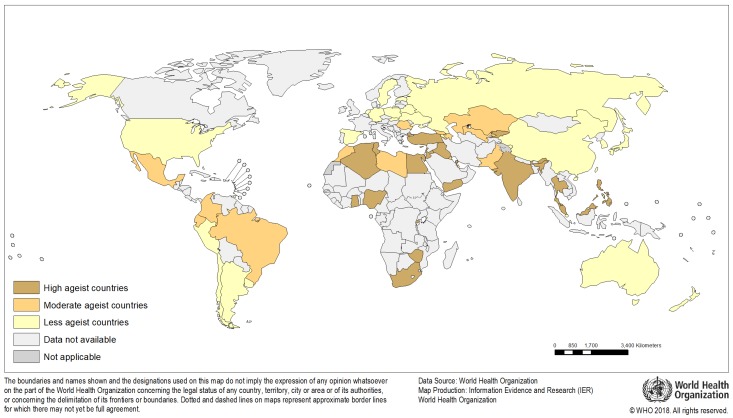
Distribution of Level 2 latent class membership across 57 countries.

**Figure 4:**
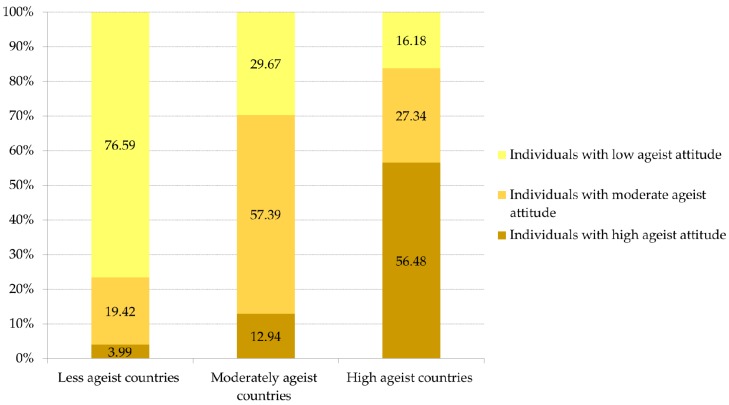
Distribution of individual-level latent class membership by Level 2 latent class.

From the 57 countries included, 34 were classified as moderately or highly ageist. Figure 3 shows the countries’ classification following the multi-level latent class analysis. The distribution of the three classes of individuals in the three classes of countries is shown in Figure 4.

### 3.3. Individual-Level and Country-Level Covariate Effects

After controlling for all individual- and country-level predictors, the results showed that being less educated, younger and male significantly increased the odds of an individual having high ageist attitudes (Table 1). Participants’ characteristics also had an effect on the probability of an individual having moderate ageist attitudes, though after controlling for country-level predictors, only the effect of education remained statistically significant.

In the mutually adjusted models, two country-level predictors—HLE and proportion of older people—remained strongly associated with high and moderate ageist attitudes of individuals. A one-year increase in HLE was associated with a lower proportion of individuals having high and moderate ageist attitudes. Similarly, a one percent increase in the proportion of older people aged over 60 years was associated with a lower proportion of individuals exhibiting high and moderate ageist attitudes.

At the country level (Table 2), HLE and the proportion of people aged over 60 years were significantly and negatively associated with the likelihood of a country being highly and moderately ageist in the mutually adjusted model. A one-year increase in HLE reduced the probability of a country being highly ageist by 27% and also reduced the probability of a country being moderately ageist by 9%. A one-unit increase in the proportion of people over 60 reduced the probability of a country being highly ageist by 32% and moderately ageist by 26%.

## 4. Discussion

Ascertaining the global prevalence of ageism and associated factors is a necessary step in understanding the magnitude of this public health issue. This study used the largest sample to date to examine ageism and its relation to various personal, demographic and development factors, including sex, age, education and healthy life expectancy (HLE).

This study shows that ageism is highly prevalent across the world, with the highest prevalence observed in poorer countries. Individuals with high ageist attitudes were more likely to reside in low- and lower-middle-income countries, such that low- and lower-middle-income countries were five times more likely to be ageist than high-income countries. The large heterogeneity observed in the meta-analysed estimates of individual-level ageist attitudes by country reflects the true variation in the prevalence of ageism across countries. In the multi-level analysis, we found that contextual factors did explain the source of variance in prevalence.

Our study also shows that two country-level factors are significantly associated with the presence of individuals with ageist attitudes and ageist countries: lower HLE and fewer people over the age of 60 as a proportion of the total population. In view of the available literature, it is possible that the relationship between HLE and ageism is bi-directional with lower levels of HLE resulting in increased ageism, and increased ageism resulting in lower levels of HLE. Countries with a lower HLE are more likely to have older adults in poor health, and increasing the populations’ exposure to poor health in older age is likely to reinforce negative attitudes towards getting older. Indeed, negative attitudes towards older age have been traced back to people’s assumption that there is an age-related decline in people’s functioning [22].

In turn, holding ageist attitudes at an individual level can decrease health and functioning. Self-directed ageism is associated with an increased cardiovascular response to stress [23], a higher presence of markers of Alzheimer’s disease [24], lower physical and cognitive function [11], and worse health behaviours [25,26]. Even the threat of stereotypes, raised by explicitly comparing an older person with younger people, is sufficient to reduce older people’s mathematical and cognitive performance by as much as 50% [27]. Longitudinal research from the United States also found that older people who hold negative attitudes towards ageing live on average 7.5 years less (after controlling for key determinants) than people with positive attitudes to ageing [28]. This supports previous work that found that being dissatisfied with ageing was a good predictor of mortality [29].

The second country-level factor significantly associated with ageism is the percentage of people aged 60 and over. In keeping with other research, these findings show that levels of ageism are lower when there is a greater proportion of older adults in a country [12]. Currently, higher-income countries have a larger percentage of older adults than low- and middle-income countries, but this is changing rapidly. For example, while France had almost 150 years to adapt to a change from 10% to 20% in the proportion of the total population aged 60 years and over, countries like Brazil and China will have slightly more than 20 years to make the same adaptation [2]. The association found between population ageing and ageism appears to indicate that as the proportion of older adults increases, members of society become more favourable towards older age. A plausible explanation is that this demographic shift offers increased opportunities for intergenerational contact, which exposes younger people to counter-stereotypical examples that can help challenge the negative associations they hold about older adults [30]. Indeed, when intergenerational contact meets certain conditions, such as frequency and reciprocity, it is associated with a decrease in negative attitudes [31]. Future research will be needed to understand if the increase in the proportion of adults over 60 years of age in low- and middle-income countries will result in lower levels of ageism, given that the rapid pace of population ageing in these countries may not offer sufficient time to adapt to the demographic shift and foster positive intergenerational contact, as suggested by North and Fiske [14]. The influence of these two country-level factors on ageism may help explain the increased levels of ageism found in low- and lower-middle-income countries as these countries tend to have both lower HLE and a lower proportion of older adults.

At the level of the individual, this study found a significant association between higher levels of education and lower levels of ageism. This finding is supported by research on attitudes across cultures showing that more educated populations are less likely to have negative perceptions of older people [15]. Other cross-cultural studies found that more educated people consider “old age” to start later, do not self-identify as an older person and hence immunise themselves against self-directed ageism [22]. Attaining a higher level of education has also been correlated with the acknowledgement of age bias and a greater sensitivity to ageism as a social problem [15].

Being older was also significantly associated with a decreased risk of exhibiting high ageist attitudes but was not significantly associated with a decreased risk of exhibiting moderate ageist attitudes. Results from past research have been mixed. One previous study found that older adults had lower explicit preferences for younger people [30], while another showed that older adults tend to be more ageist towards those older than themselves and that middle-aged people are more ageist than both younger and older groups [32]. In view of our findings and the mixed results obtained in past studies looking into this association, this is an area that would benefit from additional research.

Lastly, this study found that being male is significantly associated with the presence of high ageist attitudes at an individual level. These findings are consistent with other studies that have found that men, across all age groups, are more ageist towards older adults than women [32] and that women generally identify more with their age and view older adults as contributing more to the economy [15].

In reviewing the findings, it is important to consider the limitations of the study. While this study covered two dimensions of ageism (i.e., stereotypes and prejudice), we were unable to capture the third dimension of ageism (i.e., discrimination), as this dimension is not included in the WVS. Additionally, our findings are only as valid as the availability of individual-level indicators and the reliability of the original data sources. As data for many of the World Bank and exposure variables are available only for certain years, covariate and outcome data are not optimally time-matched for all countries. However, we do not regard this as a major threat to the validity of our findings because national-level indicators change slowly. Finally, it is important to note that we do not claim causality for any of the correlations presented here; many potential variables might affect both ageism and our exposures of interest.

## 5. Conclusions

This study used the largest sample to date to examine ageism and revealed that it is highly prevalent across individuals and countries. Ageism, despite its magnitude and negative impact on the health and functioning of older people, is not yet considered a public health priority. The findings of this study strengthen the case for a Global Campaign to Combat Ageism [3], including investments in interventions that have already been found to be effective for reducing ageism [33]. This study also reveals areas where future research is needed. For example, the cross-sectional nature of this study did not allow examining changing attitudes over time, which may be possible if further waves of the WVS repeat the questions included in this study. Understanding trends in ageist stereotypes and prejudice, and their relation to specific interventions or changes in demography and HLE are important future directions. Research is also needed to increase understanding of the directions of the correlations found in this study.

## Figures and Tables

**Table 1:** Multinomial logistic regression results: influence of individual characteristics and contextual factors on Level 1 latent class membership.

Predictors		Comparison: High Ageist Attitude vs. Low Ageist Attitude			Comparison: Moderate Ageist Attitude vs. Low Ageist Attitude		
		Model 1 ^a^	Model 2 ^b^	Model 3 ^c^	Model 1 ^a^	Model 2 ^b^	Model 3 ^c^
		Odds Ratio (95% CI)	Odds Ratio (95% CI)	Odds Ratio (95% CI)	Odds Ratio (95% CI)	Odds Ratio (95% CI)	Odds Ratio (95% CI)
Individual characteristics							
Age		0.97 (0.96–0.98) **	-	0.98 (0.97–0.99) **	0.98 (0.97–0.99) **	-	1.0 (0.99–1.01)
Sex	Female	Ref	-	Ref	Ref	-	Ref
	Male	1.2 (1.1–1.3) **	-	1.2 (1.1–1.3) **	1.2 (1.1–1.3) **	-	1.0 (0.96–1.2)
Education	None or incomplete primary	Ref	-	Ref	Ref	-	Ref
	Complete primary	0.46 (0.43–0.50) **	-	0.90 (0.84–0.97) **	0.55 (0.51–0.59) **	-	0.84 (0.78–0.90) **
	At least some secondary	0.27 (0.25–0.29) **	-	0.61 (0.57–0.67) **	0.47 (0.44–0.51) **	-	0.79 (0.74–0.84) **
	University education	0.18 (0.17–0.20) **	-	0.57 (0.53–0.61) **	0.37 (0.35–0.39) **	-	0.70 (0.66–0.74) **
Income (higher)		1.2 (0.99–1.4)	-	-	1.1 (0.87–1.38)	-	-
Contextual factors							
Healthy life expectancy		-	0.56 (0.43–0.73) **	0.87 (0.82–0.93) **	-	0.70 (0.54–0.90) **	0.91 (0.86–0.97) **
Population health status as self-reported		-	1.0 (0.98–1.02)	-	-	1.01 (0.99–1.03)	-
Income inequality		-	1.02 (0.99–1.03)	-	-	1.01 (0.98–1.04)	-
Proportion of older people		-	0.21 (0.20–0.22) **	0.32 (0.30–0.34) **	-	0.45 (0.44–0.46) **	0.52 (0.51–0.53) **

^a^ mutually adjusted without country-level predictors. ^b^ adjusted for Level 1 predictors. ^c^ mutually adjusted for Level 1 and Level 2 predictors. ** *p* value < 0.001.

**Table 2:** Multinomial logistic regression results: influence of contextual factors on Level 2 latent class membership.

Predictors	Comparison: Highly Ageist Countries vs. Not Very Ageist Countries		Comparison: Moderately Ageist Countries vs. Not Very Ageist Countries	
	Model 1 ^a^	Model 2 ^b^	Model 1 ^a^	Model 2 ^b^
Contextual factors	Odds ratio (95% CI)	Odds ratio (95% CI)	Odds ratio (95% CI)	Odds ratio (95% CI)
Healthy life expectancy	0.55 (0.42–0.73) **	0.73 (0.71–0.74) **	0.70 (0.54–0.90) **	0.91 (0.90–0.92) **
Proportion of older people	0.72 (0.71–0.73) **	0.68 (0.67–0.69) **	0.60 (0.59–0.61) **	0.74 (0.73–0.75) **
Population health status (self-reported)	0.97 (0.971–0.972) **	1.0 (0.80–1.02)	0.94 (0.93–0.95) **	1.0 (0.9–1.03)
Income inequality	0.97 (0.88–1.05)	-	0.97 (0.89–1.04)	-

^a^ adjusted for Level 1 predictors. ^b^ mutually adjusted for Level 1 and Level 2 predictors. ** *p* value < 0.05.

## Supplementary Materials

The following are available online at https://www.mdpi.com/1660-4601/17/9/3159/s1, Supplementary A: Statistical analysis and model selection, Table S1: Descriptive statistics of individual and country level predictors, Table S2: Fit criteria for different model specifications, Figure S1: Profile plot: Level 1 latent class, Figure S2: Random effect estimates for Latent Class 1 (high ageist attitude) by high income countries, Figure S3: Random effect estimates for Latent Class 2 (moderate ageist attitude) by high-income countries, Figure S4: Random effect estimates for Latent Class 3 (low ageist attitude) by high-income countries, Figure S5: Random effect estimates for Latent Class 1 (high ageist attitude) by upper middle income countries, Figure S6: Random effect estimates for Latent Class 2 (moderate ageist attitude) by upper middle-income countries, Figure S7: Random effect estimates for Latent Class 3 (less ageist attitude) by upper middle-income countries, Figure S8: Random effect estimates for Latent Class 1 (high ageist attitude) by lower middle-income and low-income countries, Figure S9: Random effect estimates for Latent Class 2 (moderate ageist attitude) by lower middle-income and low-income countries, Figure S10: Random effect estimates for Latent Class 3 (low ageist attitude) by lower middle-income and low-income countries.
