# Supplementary material for: Ageism, Healthy Life Expectancy and Population Ageing: How Are They Related?
Source: Int J Environ Res Public Health. 2020 May 1;17(9):3159. doi: 10.3390/ijerph17093159 (PMC7246680; doi:10.3390/ijerph17093159)
Supplement: Supplementary file 1 [file ijerph-17-03159-s001.pdf]

Ageism, Healthy Life Expectancy and Population Ageing: How Are They Related?

Alana Officer, Jotheeswaran A. Thiyagarajan, Mira L. Schneiders, Paul Nash and Vânia de la Fuente-Núñez*

Supplementary Appendix A

Statistical analysis and model selection

All statistical analysis was performed using Mplus version 8. We performed Multilevel latent class analysis (MLCA) incrementally to extract homogeneous latent classes of individuals based on their responses to nine ageism items (World Values Survey questions). Based on the distribution of individual-level latent classes within countries, distinct latent classes of countries were identified to classify countries as low, moderate and highly ageist. Because our latent class (LC) items of ageism were order-categorical items, we employed nonparametric estimation not assuming normality. We used full-information maximum likelihood (FIML) estimation — which allows for dependent variable missing data under missing at random (MAR) assumptions with the robust maximum likelihood estimator (MLR).

To identify the best-fitting model, we used the four stage sequential modeling strategy proposed by Henry & Muthen (2010). In the first stage, we ignored the multilevel structure of the data and estimated a series of traditional LC models to determine the number of latent classes at the individual-level. In the second stage we estimated a series of MLCA models to account for the multilevel structure of the data. In these models, the number of individual-level classes was based on the best fitting LC analysis model from the previous stage, and the LC model at the individual-level was estimated to identify the number of country-level LCs.

Model fit of the competing models was compared using the Bayesian Information Criterion (BIC), where lower values indicate better model fit to the data. The BIC is the preferred measure for simultaneously deciding about the number of lower- and higher-level classes in multilevel mixture model. Classification quality of the competing models was assessed using entropy, a measure that summarizes how well the latent classes can be distinguished. Entropy values range from 0 to 1, with higher values indicating clearer distinctions among the latent classes. In addition, the mean class assignment probabilities equal to or larger than 0.8 was considered as a good class solution. At each stage, a parsimonious solution (one of more small classes) was considered in selecting a model with fewer classes. Additionally, models were evaluated and compared according to interpretability of the obtained solutions. A theoretically meaningful solution was preferred to uninterpretable solutions. The effect of covariates on latent class membership was examined in multinomial logistic regression. As a first step, each covariate (at individual and country levels) was tested in univariate analysis. Second, covariates that achieved statistical significance in univariate analysis were included in the multivariate analysis incrementally. Countries with missing contextual factors were excluded in the regression analysis.

The latent class of ageism (measurement model)

We first performed a conventional latent class analysis wherein we ignored the nesting of individuals in countries (Supplementary Table 2, Model 1). The BIC declined (i.e., improved) when using two to four classes, after which point it began to show smaller differences. Beyond the four-class model, the quality of class separation for five-, six- and seven-class models was low; furthermore, the posterior probabilities of these classes were poor as compared to the other classes (less than 0.80). Thus, we selected two-, three- and four-class models for further investigation.

Second, we extended this model and included a common factor (random means) on Level 2 for the two-, three-and four-class solutions (Supplementary Table 2, Model 2). The substantive interpretation of the Level 1 class for individuals in each class remained the same. As compared to the fixed effects models, there was a considerable decline in the BIC and a slight improvement in the entropy in all three solutions following the addition of the common factor (random means). At this stage, we also investigated the average latent class probabilities for individuals, as these are important indicators of the quality of a LCA solution. High values of this matrix suggest that on average, individuals are classified with high levels of certainty into their most likely latent classes. In contrast, moderate or low values indicate that there is a high degree of uncertainty surrounding the class assignments. We considered values larger than 0.8 to represent a 'good' class solution. In comparison to the four-class solution, the three-class standard remained a preferable model, as the former solution separated one of the classes from the three-class solution into two smaller groups; notably, the posterior probabilities indicated that there was substantial misclassification with regard to these two smaller classes. For example, the posterior probabilities for the three-class solution were 0.89, 0.84 and 0.91, and the posterior probabilities for the four-class solution were 0.78, 0.82, 0.88 and 0.83, with the first and second classes representing the separated classes from the three-class solution. The low posterior probabilities for these two classes indicated that the model had difficulty distinguishing between individuals in the first and second classes. However, we have included all three solutions in the subsequent analysis for comparison's sake.

Third, building on this three-class solution, we added a Level 2 latent class model based on the random means from the Level 1 latent class solution. As presented in Model 3 (Supplementary Table 2), the BIC drastically declined against the fixed and random effects three-class model following the addition of two Level 2 latent classes; however, the resulting BIC was not better than the BIC for the parametric model. Adding a third Level 2 class significantly improved the BIC with regard to the parametric (common factor) and neighbouring classes (two and four). More importantly, the substantive interpretation of the three-class solution (as described in the following section) was theoretically meaningful.

Synthesising the information from all three multilevel models presented in Supplementary Table 2, we found that the parametric approach and the inclusion of a common factor on Level 2 reduced the BIC. However, adding a second common factor to the Level 2 random means greatly improved the BIC and the quality of class separation. We also examined the neighbouring Level 1 two- and four-class non-parametric random effects models. The model with the three Level 1 classes appeared to be the best fit, as it showed a substantial decline over the model with two and four Level 1 classes, maintained a high entropy value and provided the most substantively interesting solution. As such, we selected the three-by-three-class nonparametric random effects model for further examination.

Individual (Level 1) latent classes of ageism

The item profile plot for the three-class solution is presented in Supplementary Figure 1. In this solution, the largest group (Class 1) held a low-ageist attitude and comprised 44% of the participants. On the basis of the estimated conditional response probabilities, we observed that participants who were assigned to Class 1 had fairly high probabilities of endorsing higher categories for all nine indicators of ageism. This suggests that participants in this class are more likely to report that both 30- and 70-year old bosses are acceptable, to perceive older people as friendly, to view older people as competent and to treat older people with respect. Additionally, participants in this group are more likely to disagree or strongly disagree with the arguments that older people 'get more than their fair share', that older people are burden on society, that older people have too much political influence and that companies that employ young people tend to perform better.

Class 2 maintained a moderate-ageist attitude and encompassed 32% of the study participants. As compared to Class 1, Class 2 was characterised by low endorsement probabilities of the six ageism indicators, equal endorsement probabilities for two indicators (compared to Class 1) and slightly very low probabilities for the final indicators (compared to Class 3). The smallest group, Class 3, represented a high-ageist attitude and comprised 24% of the participants. In comparison with Classes 1 and 2, participants in Class 3 had very low probabilities of endorsing the eight indicators of ageism and slightly similar endorsement probabilities for the last indicator (compared to Class 2). This means that participants in this group are much less likely to report that 30- and 70-year old bosses are equally acceptable, to perceive older people as friendly, to consider older people to be competent and to view older people with respect. Compared with those in the moderate- and low-ageist classes, individuals in this class are less likely to disagree or strongly disagree with the arguments that older people 'get more than their fair share', that older people are a burden on society and that companies that employ young people perform better. Interestingly, participants classified into this group had similar endorsement probabilities as those in Class 2 for one indicator (regarding older peoples' undue political influence).

Table S1. Descriptive statistics of individual and country level predictors .

	Contextual-level Factors						Individual-level Factors					
Country	Income level[a]	WVS Sample (n)	Population Aged 60 + (%)	Healthy Life expectancy at Birth (years)	Population Health Status Based on Self-reported Poor Health (%)	Income inequality-Gini Index (%)[b]	Age (years) Mean (SD	Min	Max	Gender (Female %)	Education (University level %)	Household Income (High Category %)
Algeria	UMI	1,200	8	66.3	34.10	NA	37.8(15.1)	18	87	49.3	30.2	19.3
Argentina	HI	1,030	15	67.6	25.90	26.3	42.6(17.5)	18	92	53.6	40.4	24.2
Armenia	LMI	1,100	15	66.9	61.00	13.9	46.1(18.1)	18	85	51.5	60.9	9.0
Australia	HI	1,477	20	71.9	22.80	17.7	46.3(17.7)	18	95	48.8	45.1	25.1
Azerbaijan	UMI	1,002	9	64.7	35.50	8.9	39.6(15.3)	18	85	50.6	78.1	12.8
Bahrain	HI	1,200	4	67.0	25.50	NA	39.3(13.9)	18	72	45	50.2	45.2
Belarus	UMI	1,535	20	65.2	64.80	10.8	45.3(17.2)	18	86	54.5	65.0	15.6
Brazil	UMI	1,486	12	65.5	29.50	38.7	42.9(16.4)	18	93	52.3	43.0	15.6
Chile	HI	1,000	15	70.5	27.20	36	43.8(16.2)	18	85	50.7	30.8	15.3
China	UMI	2,300	15	68.5	13.60	29.5	42.2(14.3)	18	75	49.2	28.1	12.5
Colombia	UMI	1,512	10	65.2	23.90	37.4	40.4(15.7)	18	82	50.4	46.8	26.6
Cyprus	HI	1,000	18	71.3	30.10	15	41.9(16.9)	17	89	50.5	51.5	22.0
Ecuador	UMI	1,202	10	67.0	25.90	30.9	39.8(16.1)	18	97	51.6	27.9	21.6
Egypt	LMI	1,523	9	62.2	42.30	14.2	38.7(15.1)	18	99	50	34.6	16.5
Estonia	HI	1,533	25	69.0	53.70	18.6	47.0(18.6)	18	93	55.1	55.9	13.7
Georgia	LMI	1,202	20	66.4	57.40	23.4	44.6(17.5)	18	85	54	69.2	7.2
Germany	HI	2,046	28	71.3	31.60	14.1	49.4(17.9)	17	95	51.1	21.6	19.0
Ghana	LMI	1,552	5	55.3	11.30	31.7	32.2(13.4)	18	82	49.5	14.3	17.3
India	LMI	1,581	9	59.6	28.50	16.1	32.3(14.3)	18	86	48.2	12.8	22.3
Iraq	UMI	1,200	5	60.0	34.20	16.1	36.6(13.3)	18	83	47.6	33.7	30.8
Japan	HI	2,443	33	74.9	47.90	13.5	50.7(16.2)	18	80	51.8	71.5	19.7
Jordan	UMI	1,200	5	65.0	19.90	20.5	39.7(15.4)	18	84	50	38.8	26.4
Kazakhstan	UMI	1,500	11	63.3	39.20	12.7	40.3(15.7)	18	88	53.2	52.9	26.8
Kuwait	HI	1,303	4	65.7	14.90	NA	36.4(11.7)	16	79	36.3	64.4	40.1
Kyrgyzstan	LMI	1,500	7	63.9	24.70	17.7	38.7(14.3)	18	89	50.9	60.9	30.8
Lebanon	UMI	1,200	12	65.7	26.00	30	38.3(14.8)	18	82	51	56.5	38.8
Libya	UMI	2,131	8	63.7	17.10	NA	33.9(11.9)	18	78	47.7	50.5	30.0
Malaysia	UMI	1,300	9	66.5	13.20	NA	40.0(13.9)	18	80	48.6	24.5	45.0

		Contextual-level Factors					Individual-level Factors					
**Country**	**Income level[a]**	**WVS Sample (n)**	**Population Aged 60 + (%)**	**Healthy Life expectancy at Birth (years)**	**Population Health Status Based on Self-reported Poor Health (%)**	**Income inequality-Gini Index (%)[b]**	**Age (years)**			**Gender (Female %)**	**Education (University level %)**	**Household Income (High Category %)**
							**Mean (SD**	**Min**	**Max**			
Mexico	UMI	2,000	10	67.4	27.30	34.6	37.4(15.1)	18	93	50.1	33.5	13.5
Morocco	LMI	1,200	8	65.1	28.30	23	37.2(13.5)	18	85	50.3	13.7	7.6
Netherlan ds	HI	1,902	24	72.2	24.10	11.6	53.3(16.4)	18	90	53.5	40.3	20.2
New Zealand	HI	841	20	71.6	18.40	NA	51.4(16.8)	18	90	57.7	71.0	43.6
Nigeria	LI	1,759	4	47.7	8.30	28.4	31.5(11.7)	18	98	48.6	23.2	27.2
Pakistan	LI	1,200	7	57.8	21.70	11.6	34.4(11.9)	18	85	48.5	16.0	33.6
Peru	UMI	1,210	10	65.7	45.70	31.9	39.4(16.4)	18	88	49.8	38.0	13.6
Philippines	LMI	1,200	7	61.1	43.50	26.8	43.0(15.4)	18	87	50	41.8	15.8
Poland	HI	966	22	68.7	38.80	17.5	47.8(18.0)	19	87	53.1	31.5	13.0
Qatar	HI	1,060	2	67.8	11.30	NA	37.7(12.9)	18	93	54.2	66.2	53.0
Romania	UMI	1,503	22	66.8	38.00	17.1	46.2(17.6)	18	85	51.9	49.7	23.2
Russia	HI	2,500	20	63.4	57.00	18.7	44.6(17.2)	18	91	54.8	46.6	9.9
Rwanda	LI	1,527	4	56.6	17.20	35.2	33.7(11.2)	18	85	50.4	31.0	28.6
Singapore	HI	1,972	17	73.9	17.90	NA	45.1(17.2)	18	89	56.9	32.8	29.7
Slovenia	HI	1,069	25	71.1	34.90	11	49.4(17.6)	18	94	57.8	34.7	18.6
South Africa	UMI	3,531	9	54.4	15.00	57.3	37.7(15.6)	16	85	51.7	45.9	30.5
Republic of Korea	UMI	1,200	13	64.0	18.70	18.4	43.7(15.4)	19	85	50.6	69.9	15.8
Spain	HI	1,189	24	72.4	24.80	23.9	46.1(18.7)	18	99	51.5	22.7	10.1
Sweden	HI	1,206	26	72.0	22.80	13.1	47.6(18.2)	18	85	50.1	52.2	27.1
Thailand	UMI	1,200	16	66.8	20.70	34	45.1(12.2)	18	85	47.7	28.5	23.8
Trinidad and Tobago	HI	999	8	63.3	25.00	21.9	45.8(17.7)	18	94	54.9	13.9	22.7
Tunisia	UMI	1,205	15	66.7	29.80	18.9	38.8(16.2)	18	87	47.4	24.3	19.0
Turkey	UMI	1,605	15	66.2	29.90	21.8	40.0(15.0)	18	86	49.3	34.5	33.8
Ukraine	LMI	1,500	20	64.1	63.40	9.2	46.6(18.0)	18	89	55	59.6	13.3
United States	HI	2,232	9	69.1	18.70	35.6	46.3(16.9)	18	93	51.5	86.4	23.0

	Contextual-level Factors						Individual-level Factors					
Country	Income level[a]	WVS Sample (n)	Population Aged 60 + (%)	Healthy Life expectancy at Birth (years)	Population Health Status Based on Self-reported Poor Health (%)	Income inequality-Gini Index (%)[b]	Age (years) Mean (SD	Min	Max	Gender (Female %)	Education (University level %)	Household Income (High Category %)
Uruguay	HI	1,000	4	67.9	19.50	24.2	44.9(18.2)	18	88	52.8	20.4	13.4
Uzbekistan	LMI	1,500	20	62.4	26.40	20.1	39.3(14.8)	18	89	61.3	16.5	35.3
Yemen	LMI	1,000	12	57.7	29.50	20.6	35.5(13.2)	18	90	50.2	22.6	10.5
Zimbabwe	LI	1,500	15	52.1	15.90	35.8	36.1(14.8)	18	92	54.4	12.3	16.7

NA – Data Not Available.

Data from the following study sites were excluded from the analysis: Taiwan, Palestine, and Hong Kong.

[a]Income level: HI (high income), UMI (upper-middle income), LMI (lower-middle income), LI (low income).

[b]The Gini index is the Gini coefficient expressed as a percentage, and is equal to the Gini coefficient multiplied by 100.

Table S2. Fit criteria for different model specifications.

Models	Latent Classes		Model Fit Information			
	Individual (Level 1)	Country (Level 2)	Log-likelihood	Free parameters	BIC	Entropy
**Model 1: Fixed effect (no multilevel effect)**	2	0	-930309	65	1861354	0.73
	3	0	-919257	98	1839623	0.71
	4	0	-911651	131	1824785	0.71
	5	0	-905554	164	1812965	0.68
	6	0	-900479	197	1803188	0.68
	7	0	-895819	230	1794242	0.68
**Model 2: Parametric, with common factor (random effects)**	2	0	-925553	66	1851853	0.73
	3	0	-913009	100	1827237	0.73
	4	0	-903265	134	1808048	0.72
**Model 3: Non-parametric**	2	2	-916258	131	1833999	0.86
	3	2	-915747	101	1832638	0.81
	4	2	-888917	395	1831430	0.83
	2	3	-926299	197	1853379	0.89
	3	3	-896985	296	1797320	0.85
	4	3	-904345	139	1810263	0.83

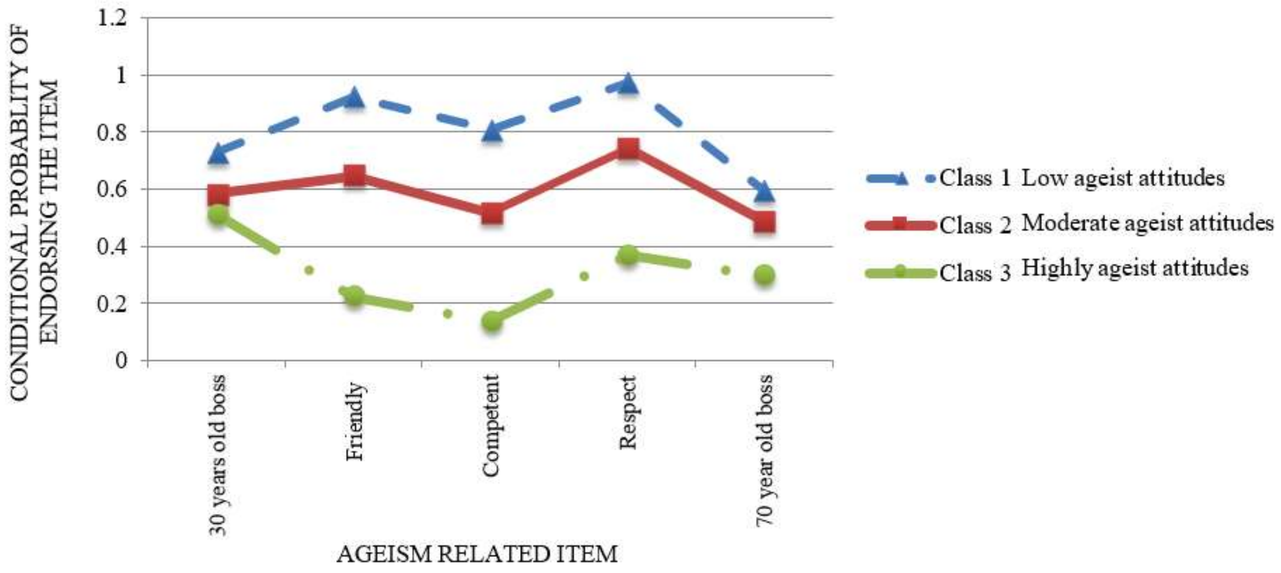

Panel A

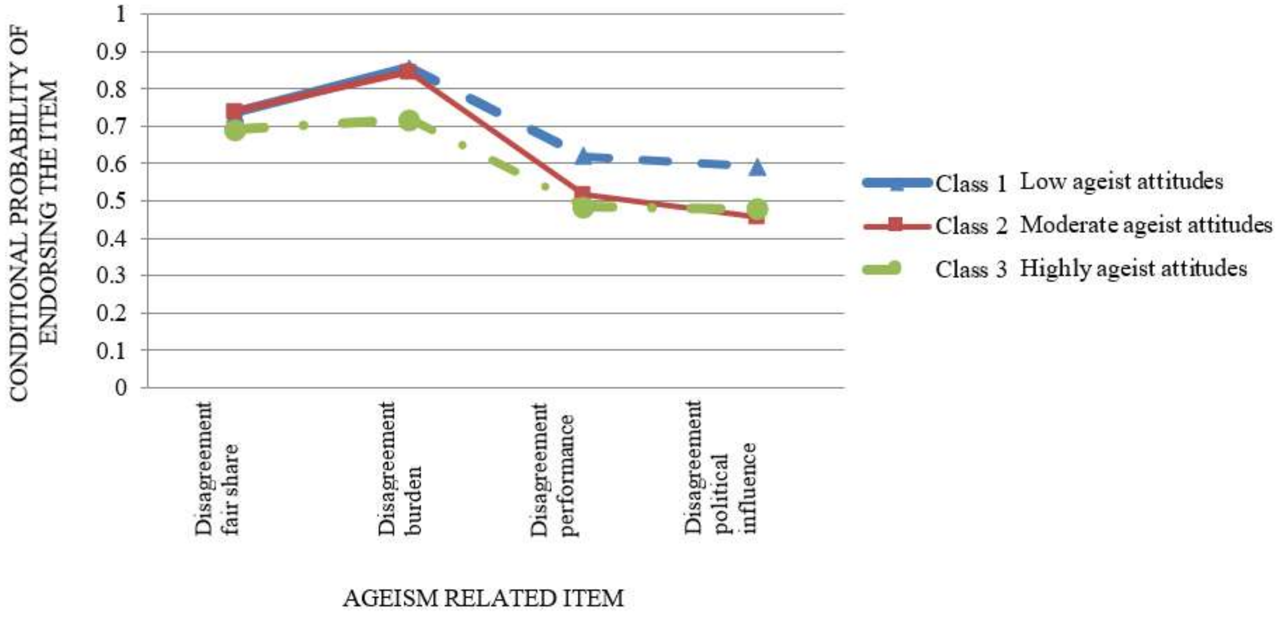

Panel B

Figure S1. Profile plot: Level 1 latent class. Panel A – conditional response probabilities for five ageism related items (asked indirectly). Panel B – conditional response probabilities for four ageism related items (asked directly).

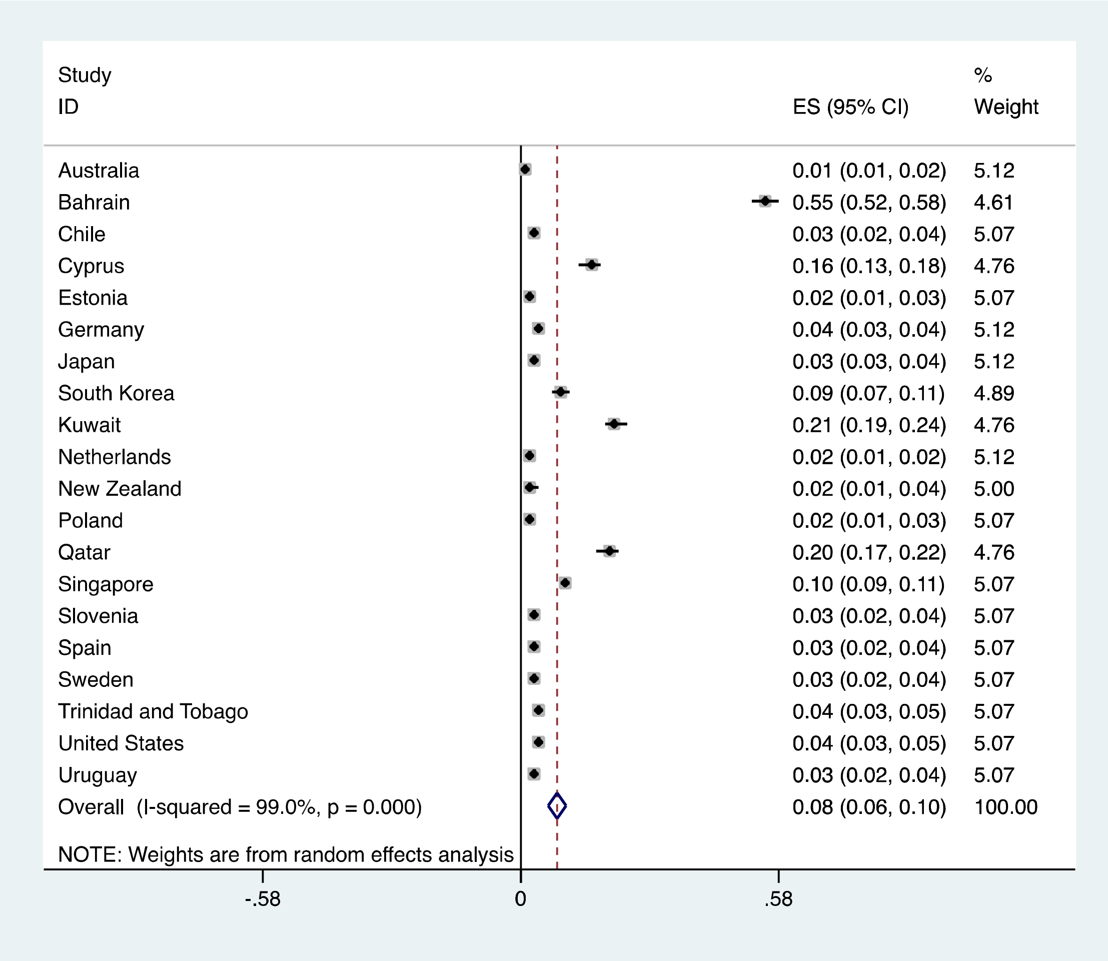

Heterogeneity calculated by formula; Q = SIGMA_i{ (1/variance_i)*(effect_i - effect_pooled)^2 }; where variance_i = ((upper limit - lower limit)/(2*z))^2 ; Heterogeneity chi-squared = 1941.15 (d.f. = 19), p = 0.000; I-squared (variation in ES attributable to heterogeneity) = 99.0%; Estimate of between-study variance Tau-squared = 0.0020; Test of ES = 0 : z = 7.99, p = 0.000

Figure S2. Random effect estimates for Latent Class 1 (high ageist attitude) by high income countries.

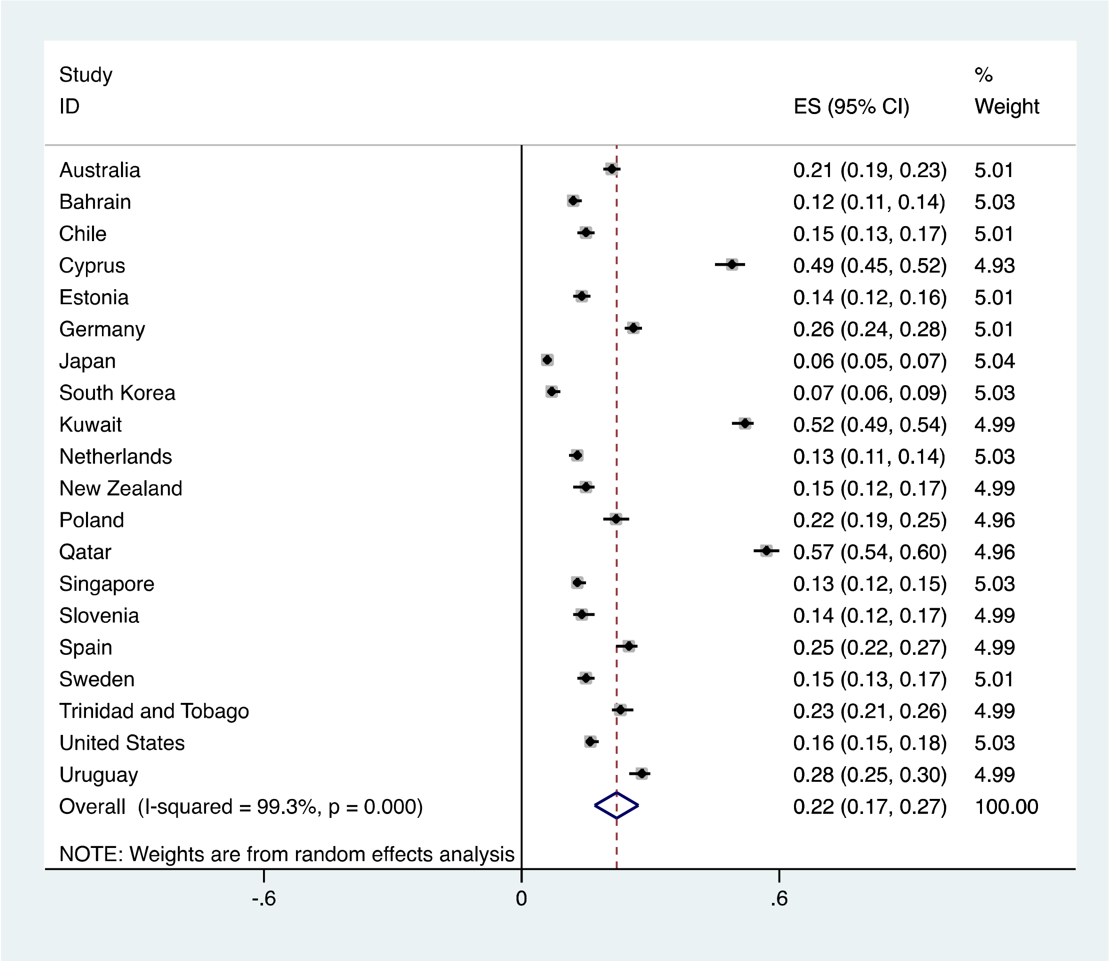

Heterogeneity calculated by formula; Q = SIGMA_i{ (1/variance_i)*(effect_i - effect_pooled)^2 }; where variance_i = ((upper limit - lower limit)/(2*z))^2; Heterogeneity chi-squared = 2746.07 (d.f. = 19), p = 0.000; I-squared (variation in ES attributable to heterogeneity) = 99.3%; Estimate of between-study variance Tau-squared = 0.0134; Test of ES=0 : z = 8.50, p = 0.000

Figure S3. Random effect estimates for Latent Class 2 (moderate ageist attitude) by high-income countries.

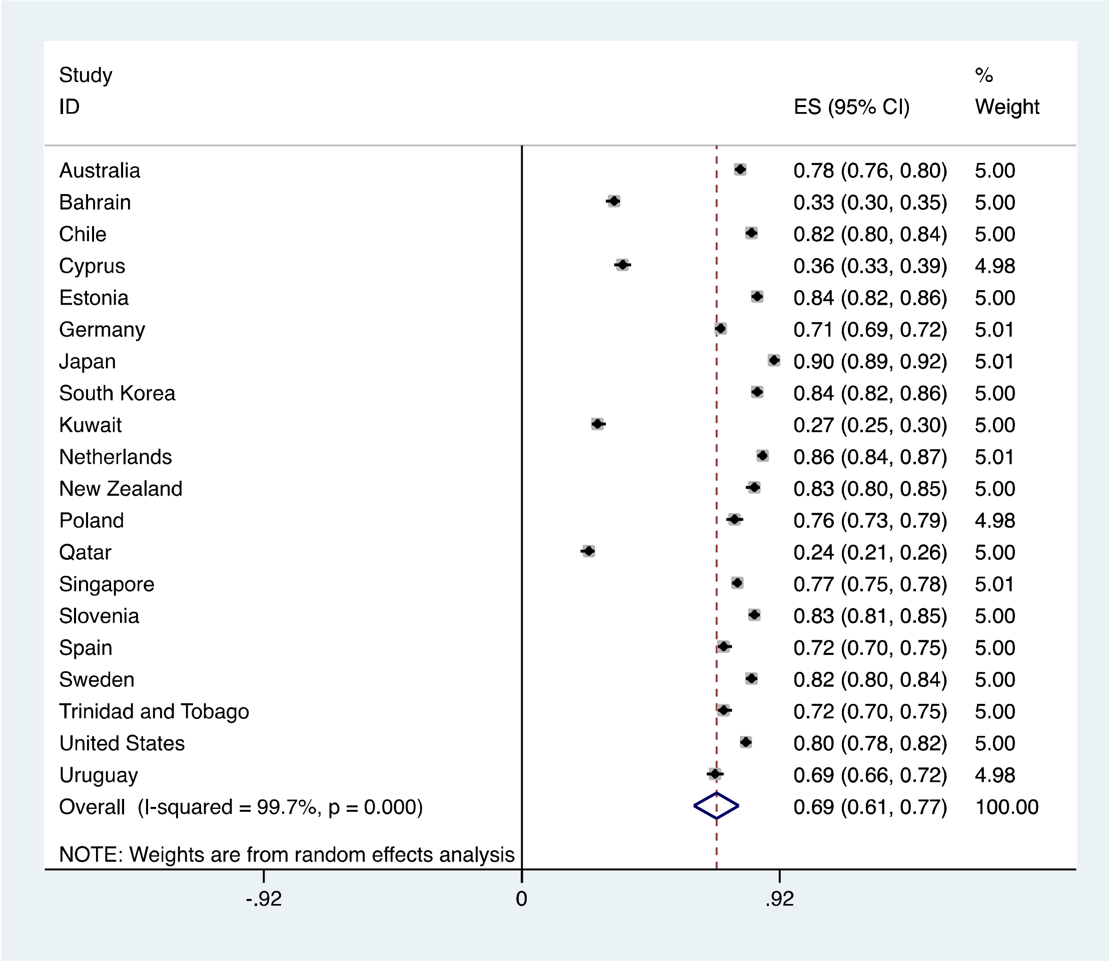

Heterogeneity calculated by formula; Q = SIGMA_i{ (1/variance_i)*(effect_i - effect_pooled)^2 }; where variance_i = ((upper limit - lower limit)/(2*z))^2; Heterogeneity chi-squared = 5767.21 (d.f. = 19), $p = 0.000$; I-squared (variation in ES attributable to heterogeneity) = 99.7%; Estimate of between-study variance Tau-squared = 0.0331; Test of ES=0 : z= 17.05, $p = 0.000$

Figure S4. Random effect estimates for Latent Class 3 (low ageist attitude) by high-income countries.

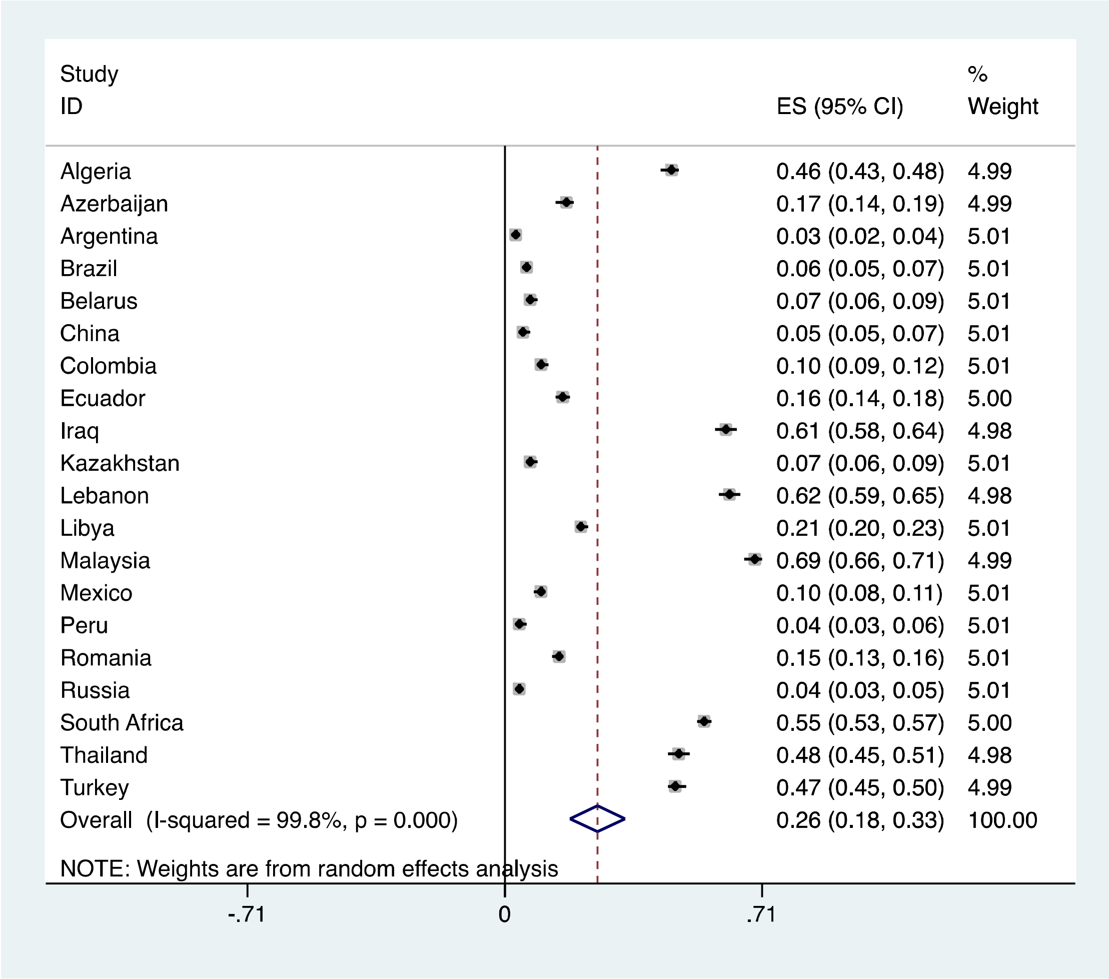

Heterogeneity calculated by formula; Q = SIGMA_i{ (1/variance_i)*(effect_i - effect_pooled)^2 }; where variance_i = ((upper limit - lower limit)/(2*z))^2; Heterogeneity chi-squared = 9078.63 (d.f. = 19), p = 0.000; I-squared (variation in ES attributable to heterogeneity) = 99.8%; Estimate of between-study variance Tau-squared = 0.0298; Test of ES = 0 : z = 6.63, p = 0.000

Figure S5. Random effect estimates for Latent Class 1 (high ageist attitude) by upper middle income countries.

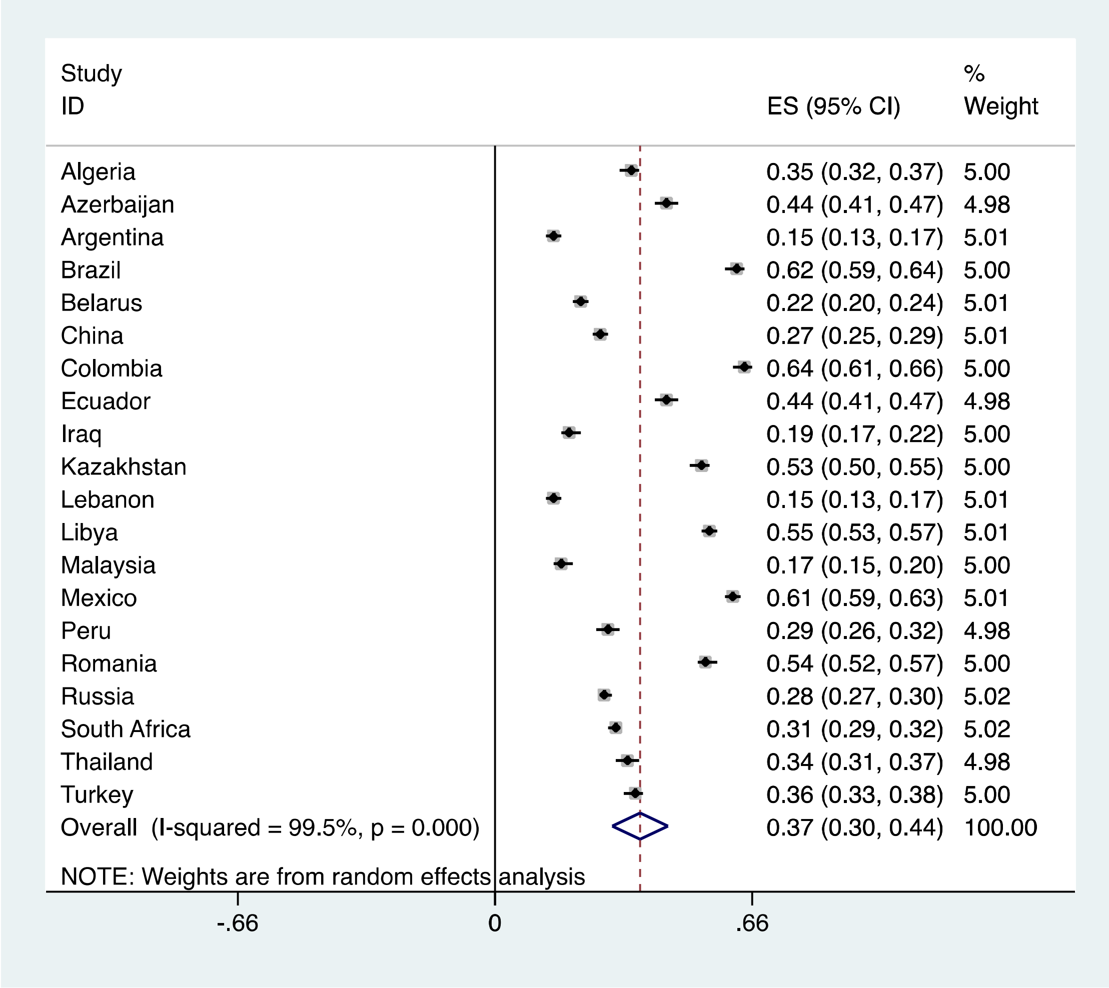

Heterogeneity calculated by formula; Q = SIGMA_i{ (1/variance_i)*(effect_i - effect_pooled)^2 }; where variance_i = ((upper limit - lower limit)/(2*z))^2; Heterogeneity chi-squared = 3959.97 (d.f. = 19), p = 0.000; I-squared (variation in ES attributable to heterogeneity) = 99.5%; Estimate of between-study variance Tau-squared = 0.0265; Test of ES=0 : z= 10.19, p = 0.000

Figure S6. Random effect estimates for Latent Class 2 (moderate ageist attitude) by upper middle-income countries.

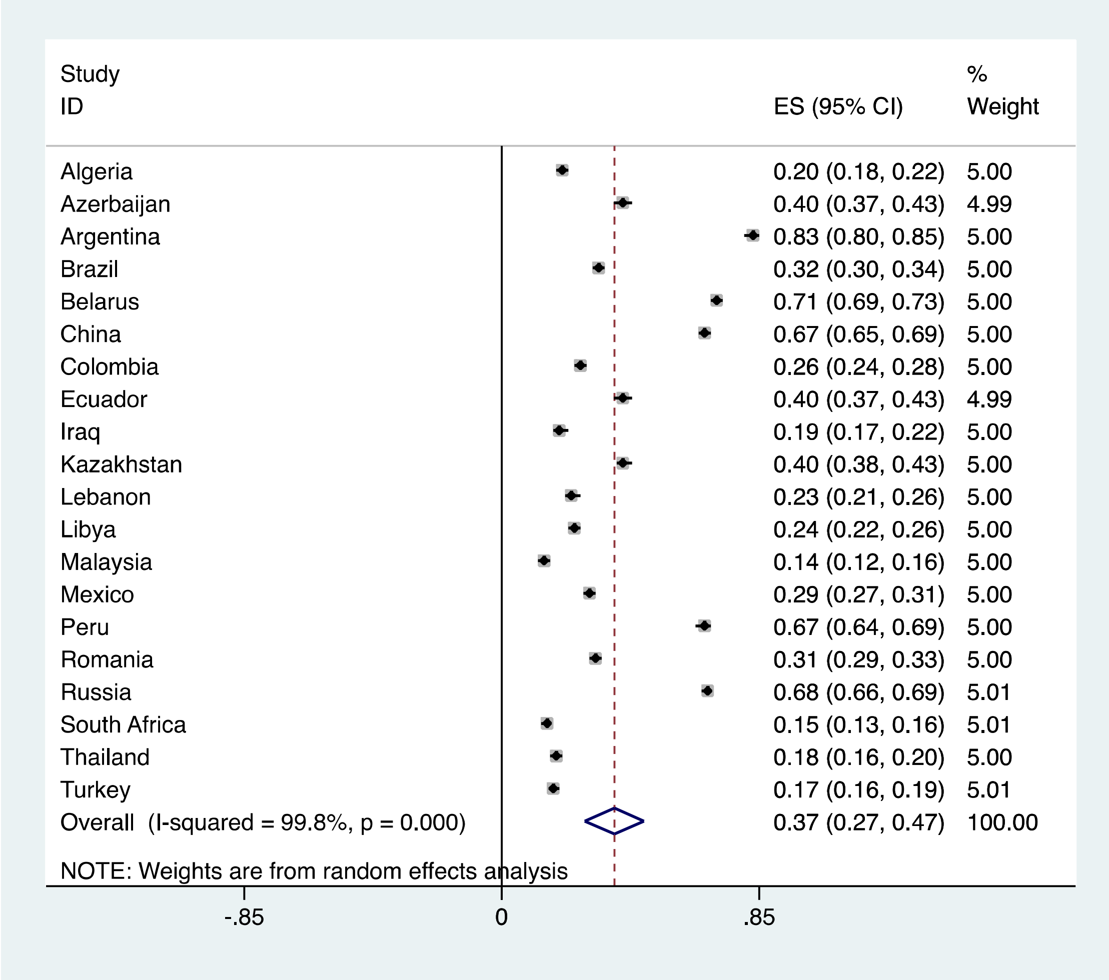

Heterogeneity calculated by formula; Q = SIGMA_i{ (1/variance_i)*(effect_i - effect_pooled)^2 } where variance_i = ((upper limit - lower limit)/(2*z))^2; Heterogeneity chi-squared = 8811.45 (d.f. = 19), *p* = 0.000; I-squared (variation in ES attributable to heterogeneity) = 99.8%; Estimate of between-study variance Tau-squared = 0.0500; Test of ES = 0 : z = 7.43, *p* = 0.000

Figure S7. Random effect estimates for Latent Class 3 (less ageist attitude) by upper middle-income countries.

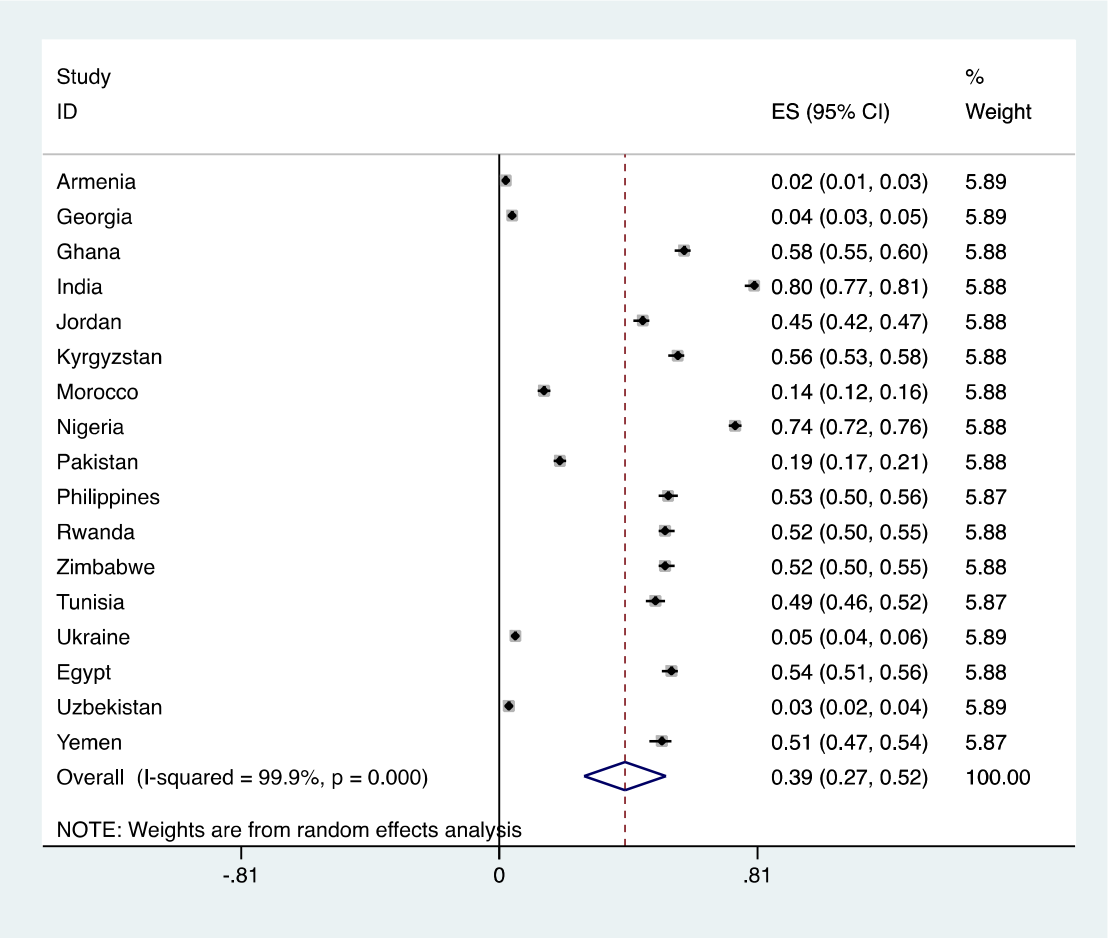

Heterogeneity calculated by formula; Q = SIGMA_i{ (1/variance_i)*(effect_i - effect_pooled)^2 } where variance_i = ((upper limit - lower limit)/(2*z))^2; Heterogeneity chi-squared = 15656.74 (d.f. = 16), *p* = 0.000; I-squared (variation in ES attributable to heterogeneity) = 99.9%; Estimate of between-study variance Tau-squared = 0.0733; Test of ES=0 : z = 6.00, *p* = 0.000

Figure S8. Random effect estimates for Latent Class 1 (high ageist attitude) by lower middle-income and low-income countries.

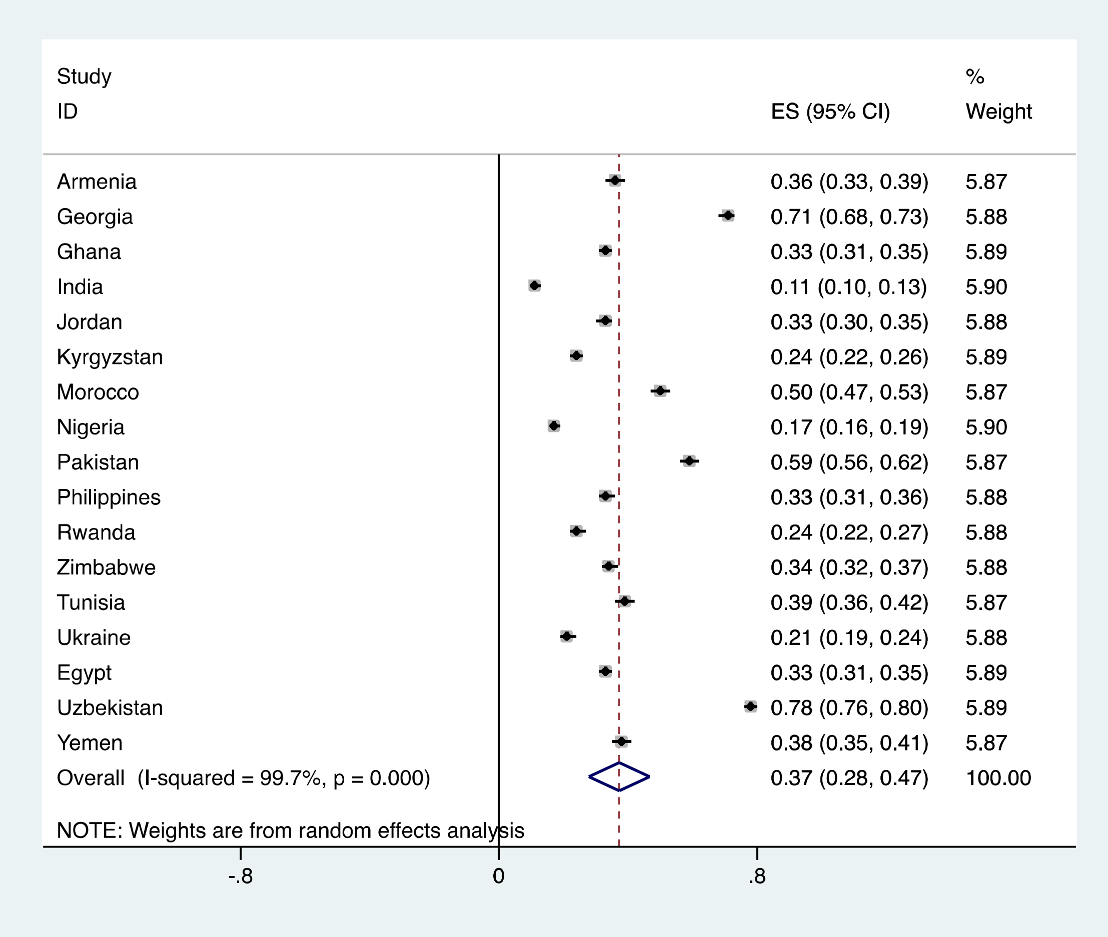

Heterogeneity calculated by formula; Q = SIGMA_i{ (1/variance_i)*(effect_i - effect_pooled)^2 }; where variance_i = ((upper limit - lower limit)/(2*z))^2; Heterogeneity chi-squared = 4752.17 (d.f. = 16), p = 0.000; I-squared (variation in ES attributable to heterogeneity) = 99.7%; Estimate of between-study variance Tau-squared = 0.0391; Test of ES = 0 : z = 7.76, p = 0.000

Figure S9: Random effect estimates for Latent Class 2 (moderate ageist attitude) by lower middle-income and low-income countries

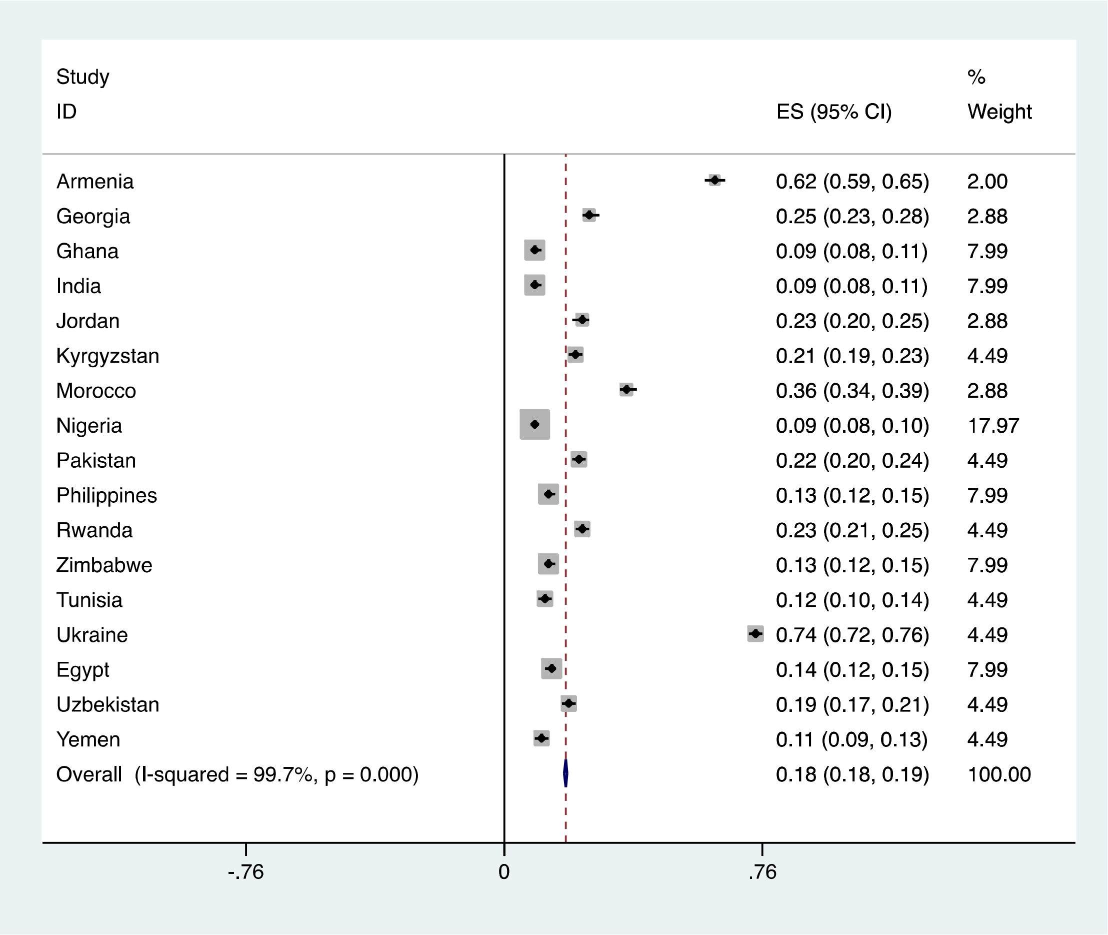

Heterogeneity calculated by formula; Q = SIGMA_i{ (1/variance_i)*(effect_i - effect_pooled)^2 } where variance_i = ((upper limit - lower limit)/(2*z))^2; Heterogeneity chi-squared = 4913.56 (d.f. = 16), p = 0.000; I-squared (variation in ES attributable to heterogeneity) = 99.7%; Test of ES = 0 : z = 83.60, p = 0.000

Figure S10. Random effect estimates for Latent Class 3 (low ageist attitude) by lower middle-income and low-income countries.
